# Tomographic reconstruction from planar thermal imaging using convolutional neural network

**DOI:** 10.1038/s41598-022-06076-z

**Published:** 2022-02-11

**Authors:** Daniel Ledwon, Agata Sage, Jan Juszczyk, Marcin Rudzki, Pawel Badura

**Affiliations:** Faculty of Biomedical Engineering, Silesian University of Technology, Roosevelta 40, 41-800 Zabrze, Poland

**Keywords:** Biomedical engineering, Diagnosis

## Abstract

In this study, we investigate perspectives for thermal tomography based on planar infrared thermal images. Volumetric reconstruction of temperature distribution inside an object is hardly applicable in a way similar to ionizing-radiation-based modalities due to its non-penetrating character. Here, we aim at employing the autoencoder deep neural network to collect knowledge on the single-source heat transfer model. For that purpose, we prepare a series of synthetic 3D models of a cylindrical phantom with assumed thermal properties with various heat source locations, captured at different times. A set of planar thermal images taken around the model is subjected to initial backprojection reconstruction, then passed to the deep model. This paper reports the training and testing results in terms of five metrics assessing spatial similarity between volumetric models, signal-to-noise ratio, or heat source location accuracy. We also evaluate the assumptions of the synthetic model with an experiment involving thermal imaging of a real object (pork) and a single heat source. For validation, we investigate objects with multiple heat sources of a random location and temperature. Our results show the capability of a deep model to reconstruct the temperature distribution inside the object.

## Introduction

The local imbalances in body temperature frequently indicate various medical disorders. Changes observed in skin surface temperature may signal physiological mechanisms leading to deviations from homeostasis, including tumors, injuries, inflammations in underlying tissues, or abnormalities in blood circulation^1^. Infrared thermal (IR) images can reflect abnormal heat distribution related to pathologies. So far, the most popular form of medical thermal imaging is the two-dimensional approach^2^. The images reflect the temperature distribution on a surface, hence only rough localization of the heat source is possible. The effects observable in thermal imaging result from complex metabolic processes associated with heat generation, so the 2D approach brings only limited information. Extending the conventional thermal imaging with the information related to internal and spatial heat distribution can support the medical diagnosis. That leads us to the concept of 3D thermal tomography for the development of non-invasive imaging.

Heat transfer within living tissues constitutes a complex process. The intricacy comes from considering various biological aspects (blood perfusion, metabolism, etc.) required to describe the issue accurately. Researchers proposed several bioheat transfer models^3,4^. The Pennes bioheat equation is one of the best known and most common approaches to present the temperature distribution of a tissue as a function of metabolic and blood perfusion rate^4–6^. It considers multiple parameters, e.g., the mass density, thermal conductivity of the tissue, blood density, specific heat, or perfusion rate. Various types of testing phantoms have been employed by heat distribution research. Depending on the scientific purposes, they differ in materials, dimensions, and shapes. Researchers willingly use cylindrical models, especially for preliminary studies, due to their noncomplex structure^7–9^. Sadeghi et al.^7^ reported the use of a cylindrical container filled with temperature-controllable gel placed on a heating plate. However, the authors presented only practical experiments, while they ignored mathematical modeling. Lim et al.^8^ also employed the cylinder-shaped model, and the mathematical calculations involved the Pennes equation. They used temperature distribution to demonstrate thermal treatment effects of electromagnetic focusing gained with a phase compensation technique for microwave hyperthermia systems. The cylindrical phantom and Pennes bioheat equation were also used by Kim et al.^9^. The authors proposed a dual-purpose patch antenna for MRI imaging and RF heating hyperthermia and evaluated the temperature distribution during their setup.

3D thermal imaging techniques described in the literature generally embrace three categories: (1) combination of 2D IR imaging and depth data^10–14^, (2) reconstruction based on sensors attached to the object surface^15^, and (3) reconstruction from a series of 2D IR images (thermal tomography)^16,17^. The first group returns the spatial shape and surface temperature distribution, while the other two address the temperature distribution within the object. Landmann et al.^10^ reported a method that combined 2D IR data and depth data in the form of a high-speed acquisition tool constructed of the light sensor and long-wave IR camera. The system returns a 3D model with temperature data on its surface. A similar approach, merging thermal and depth data, was described by Chromy and Zalud^11^. The proposed RoScan consisted of an IR camera, a color camera, and a laser scanner. Schollemann et al.^12^ involved a similar idea, yet 3D computed tomography volume constituted the depth data. Chen et al.^13^ proposed a 3D IR imaging approach for power systems evaluation using a series of 2D IR images acquired around the enclosed heat source. The technique employed the intersection method to reconstruct volumetric temperature data of an object.

A significant advantage of the third group of studies can be found in employing only the data generated by IR cameras. This paper falls into this category, in which only individual studies have been reported so far. Koutsantonis et al.^16^ presented a tomographic image reconstruction method in IR tomography, called RISE (Reconstructed Image from Simulations Ensemble), combining statistical physics concepts and Monte Carlo methods. The goal was to extract the physical parameters of an object and simulate a pack of image configurations corresponding to a tomographic problem solution. The process was evaluated using a simulated dataset and experimental data obtained from a thermal phantom. The method showed increased quality than two reference reconstruction methods: algebraic reconstruction technique (ART)^18^ and maximum-likelihood expectation-maximization (MLEM)^19^. However, the phantom medium employed in this study differs from the human body tissue in terms of absorbance. Sage et al.^17^ reported preliminary results on 3D thermal reconstruction from a series of 2D IR images for medical purposes. The reconstruction involved a backprojection method over an agarose phantom. The initial effects indicated the ability to distinguish areas of abnormal temperature distribution that correspond to the actual location of the heat source.

This study is a development of our research from Sage et al.^17^. For thermal tomography purposes, we employed deep learning. The capability of deep learning applied to different kinds of medical image processing and analysis has been a major issue in the last decade in terms of, e.g., image classification, object detection, semantic segmentation, or registration^20^. A review by Ben Yedder et al.^21^ lists several methods designed for medical image reconstruction using deep neural networks, yet none of them addresses IR imaging. The convolutional neural networks (CNN) application scope broadens rapidly, also in terms of the input image shape and size. The original works and models were designed for 2D images. Yet, with the consistent development of both hardware resources and implementation concepts also the analysis of 3D structures became applicable in a reasonable time. CNNs have increased attention in the field of medical image processing as the models constitute a fusion of deep learning and image processing methods^22^. The presence of convolutional layers enables the extraction of features from the image. The spatial relations are crucial for complete image understanding, and the CNN preserves them. Additionally, pooling layers guarantee the elimination of irrelevant features, hence the number of trainable parameters and computation power requirements decrease. CNNs support the control of possible overfitting and secure invariance to local translation^22–24^. For all those reasons, CNN appears as a suitable approach for infrared image processing^25^. Several studies reported the employment of recurrent neural networks (RNN) for image operations, including segmentation and classification^26–28^. However, their main application fields are text and audio processing. In contrast to CNN, the RNN primarily operates on sequential (time-domain) data. It is also considered sensitive to the exploding or vanishing gradient problems, and the extraction of image features is more robust in CNNs^26,28^. Here, we design and train a deep model (CNN) analyzing an input volume containing initial tomographic reconstruction from planar thermal images.

This paper concisely presents the novel concept of CNN-based thermal tomography and the experimental setup in both synthetic and real data with single or multiple heat sources. The original contribution brought by the study can be found in: (1) the employment of deep learning into the field of thermal tomography, (2) investigating the ability to reconstruct the heat distribution from planar thermal images, and (3) supporting synthetic design and validation of the model with the experimental setup involving a real object (pork) heated from a single source. We also assess reconstruction performance using benchmark metrics. Our results prove the capability of a deep model to reflect the temperature distribution inside the object and set the starting point for further development of the method. We expect the knowledge-based model designed and trained for thermal tomography to make an essential contribution to artificial intelligence-based computer-aided diagnosis and decision-making.

The materials and methods are described in “Materials and methods” section, including generation and validation of the synthetic data and specification of our deep model for thermal reconstruction. “Results” section presents a series of experiments over both synthetic and real data. The results and perspectives for artificial intelligence-based thermal tomography are discussed in “Discussion” section. “Conclusion” section concludes the study.

## Materials and methods

### Synthetic dataset

**Figure 1:**
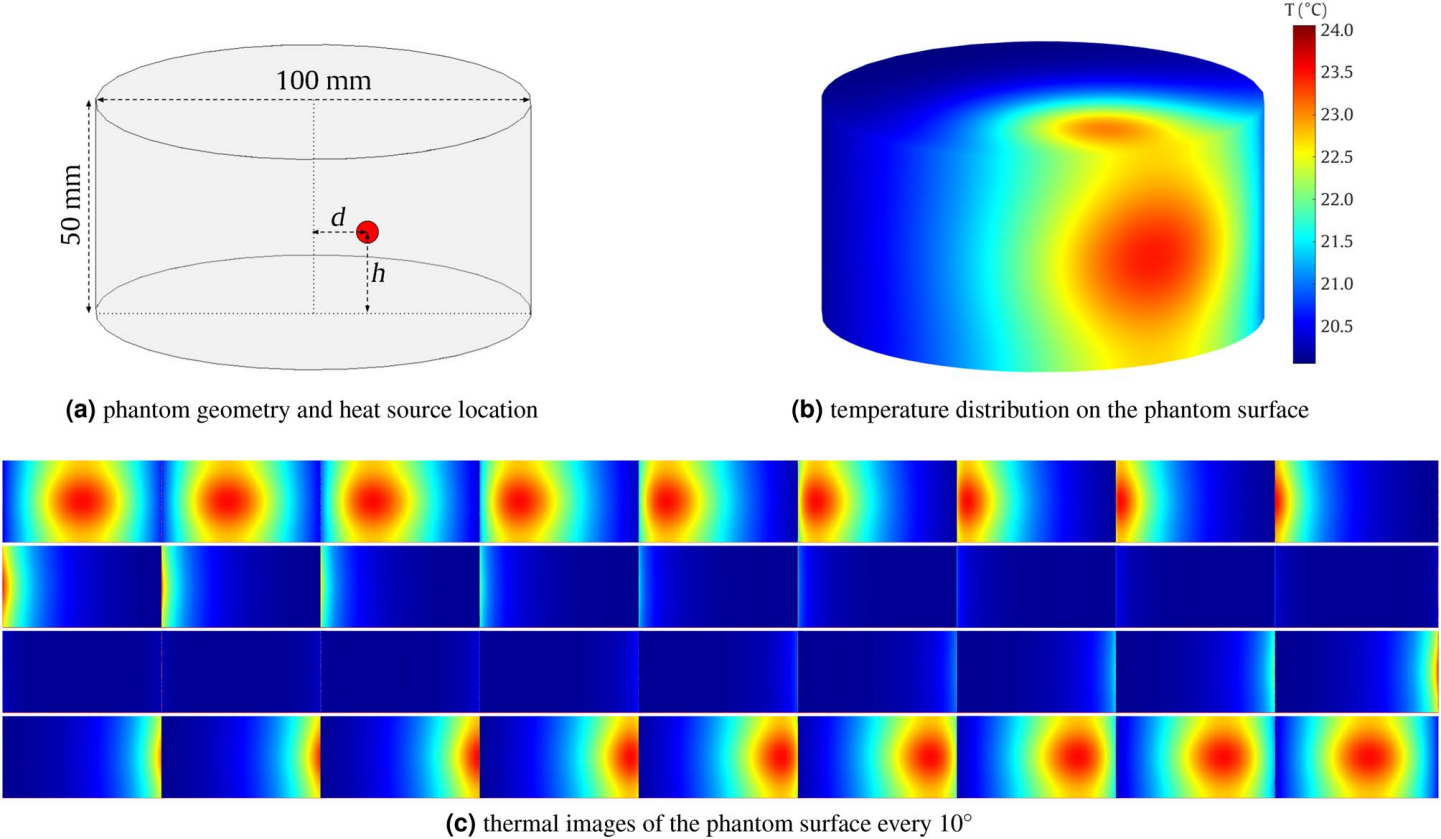
Illustration of the synthetic dataset generation from a cylindrical phantom.

The synthetic dataset was prepared using a computer simulation using MATLAB Partial Differential Equation Toolbox with the general formula:

1 $$\begin{aligned} \rho C_p \frac{dT}{dt} - \nabla \cdot \left( k\nabla T\right) = f, \end{aligned}$$

where *T* is the temperature, *k*—the thermal conductivity, $\rho$—the mass density, $C_p$ denotes the specific heat, and *f* is the heat generated inside the object ($f=0$ in our case). We created a 3D cylindrical phantom with a diameter of 10 cm and a height of 5 cm using Blender^29^ software (V2.93 Blender Foundation, https://www.blender.org/) and exported it to the .stl file (Fig. 1a). The heat source was a 0.5 cm diameter sphere with a variable location inside the phantom. The distance *d* of the heat source center from the phantom axis toward the side wall varied from 0.5 to 4.5 cm with a step of 0.5 cm. The same range was used to change the height *h* at which the heat source was placed. This allowed for 81 unique combinations of object locations within the phantom.

Prepared models of the phantom and source geometry were used in heat transfer analysis^30^. For the phantom interior, we set the thermal properties comparable to muscle tissue^31^. The exact values are given in Table 1. Instead of providing the heat source explicitly, we used the Dirichlet boundary condition with a constant temperature of 50$^{\circ }$C on the source surface during the transient analysis. In each case, the initial temperature of the phantom was 20$^{\circ }$C. The same value of ambient temperature and the radiation emissivity coefficient of 0.95 served as the boundary conditions for the phantom surface. Minkina and Dudzik^32^ have shown that two main quantities can significantly influence temperature measurement errors: object emissivity and ambient temperature, while other parameters under consideration: relative humidity, camera-to-object distance, and atmospheric temperature, do not contribute to the error. We treated the initial temperature as a reference and modeled the heat distribution in a differential manner ($\Delta T$ in relation to 20$^{\circ }$C). For high thermal contrast, the source temperature during the simulation was 50$^{\circ }$C ($\Delta T=30 ^{\circ }$C). We prepared several states of the model that resemble the heat distribution. The states were captured in 5-minute intervals from the 15th to the 60th minute from the beginning of the simulation (Fig. 1b, c). Hence, the synthetic database contained a total of 810 unique thermal models.

**Table 1:** Thermal properties of muscle tissue used in phantom heat transfer simulation.

Parameter	Symbol	Value	Unit
Thermal conductivity	*k*	0.49	W/(m $^{\circ }$C)
Mass density	$\varrho$	1103	kg/m$^3$
Specific heat	$C_p$	3322	J/(kg $^{\circ }$C)

**Figure 2:**
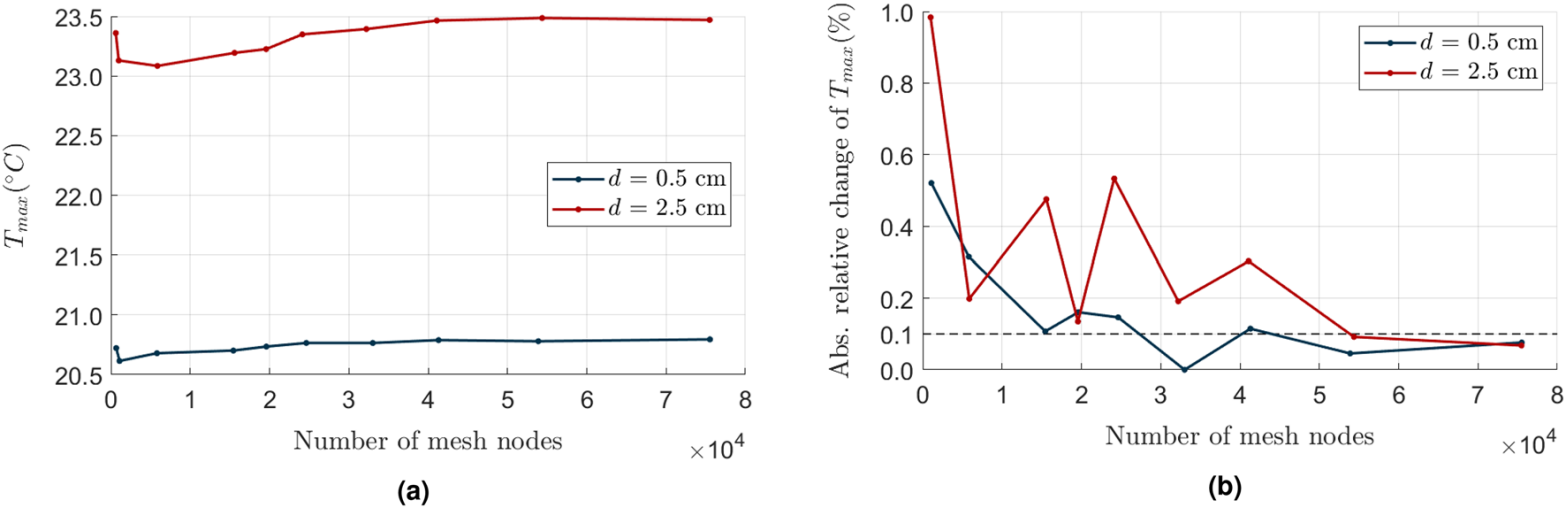
Maximum temperature of the model surface $T_{max}$ vs. the number of nodes in the mesh independence study. (**a**) shows the absolute relative changes of $T_{max}$ from (**b**) in subsequent mesh sizes. The dashed line shows the $0.1\%$ level in (**a**).

For the mesh independence study, we verified a sequence of maximum mesh edge lengths from 5 cm down to 4 mm (descending order). The assumed model geometry produced meshes consisting of ca. 500 to 75,000 nodes. To assess the mesh, we measured the maximum temperature $T_{max}$ in the object surface nodes in the 60th minute. Figure 2 presents the results of two experiments with various heat source locations (*d* = 0.5 and 2.5 cm; $h=2.5$ cm in both cases). Since the 0.1% change is considered a reliable indicator of the mesh independence^33,34^, we set the maximum mesh edge length to 0.5 cm in our study, obtaining ca. 40,000 nodes. That gave us sufficient resolution to produce projections of acceptable quality, comparable to the actual measurements with an IR camera. Denser mesh significantly increased the time consumption with a negligible impact on the quality of the model.

**Figure 3:**
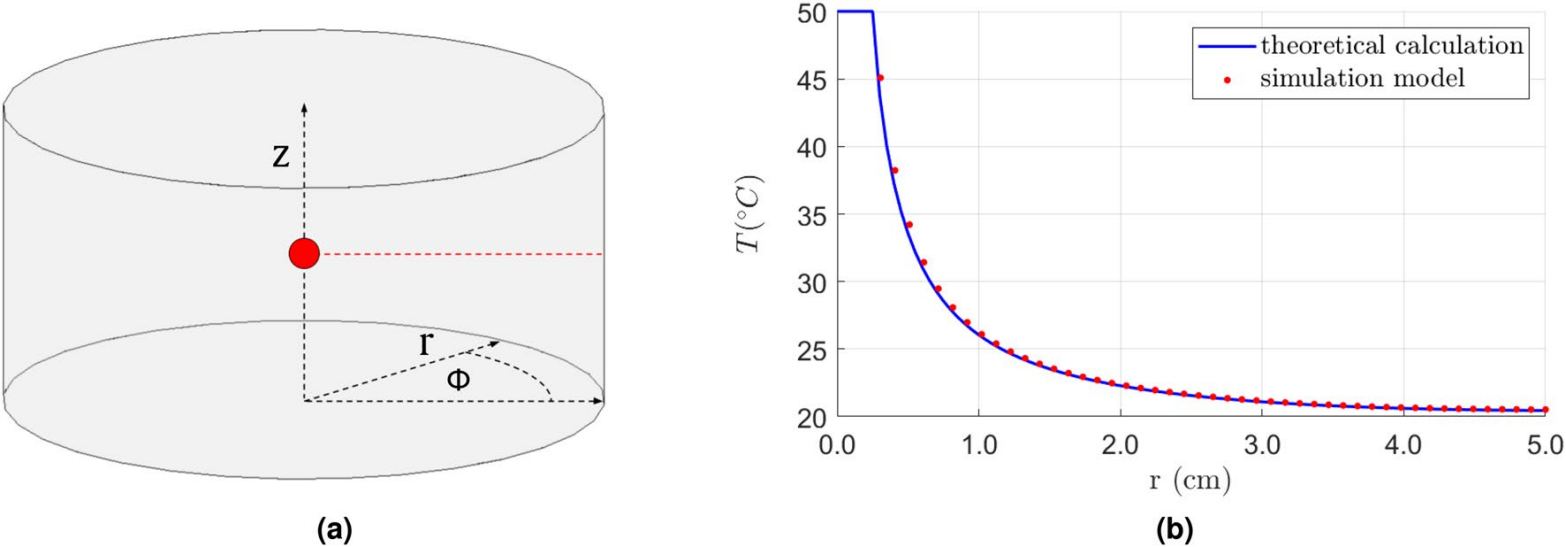
Validation of the model: illustration of the setup in the cylindrical coordinate system (**a**) and temperature distribution on the cylinder radius originating in the heat source center (red profile in (**b**), $z=h=2.5$ cm) (**b**).

We also validated the simulation for conformity with the theoretical heat transfer. We used the general heat conduction equation (1) for transient problems in a 3D space to calculate the analytical solution. Like in our simulation, we assumed a homogeneous and isotropic object with a constant thermal conductivity *k*. Considering our phantom geometry, we used the heat conduction equation in a 3D cylindrical coordinate system^35^:

2 $$\begin{aligned} \frac{\partial ^2T}{\partial r^2} + \frac{1}{r} \frac{\partial T}{\partial r} + \frac{1}{r^2} \frac{\partial ^2T}{\partial \phi ^2} + \frac{\partial ^2T}{\partial z^2} + \frac{f}{k} = \frac{1}{\alpha } \frac{dT}{dt}, \end{aligned}$$

where *r*, *z*, $\phi$ are cylindrical coordinates (Fig. 3a), and $\alpha = \frac{k}{\rho C_p}$ is the thermal diffusivity. For simplicity, we placed our spherical heat source in the middle of the cylinder ($d=0$, $h=2.5$ cm), so the temperature distribution does not depend on the $\phi$ angle. Moreover, $f=0$, as we model the heat source surface by the Dirichlet boundary condition. Therefore, Eq. (2) takes the form:

3 $$\begin{aligned} \frac{\partial ^2T}{\partial r^2} + \frac{1}{r} \frac{\partial T}{\partial r} + \frac{\partial ^2T}{\partial z^2} = \frac{1}{\alpha } \frac{dT}{dt}, \end{aligned}$$

The outer boundary condition on the cylinder surface is defined in the radiation terms:

4 $$\begin{aligned} -k \frac{\partial T}{\partial \overrightarrow{n}} = \varepsilon \sigma \left( T^4 - T_{\infty }^4 \right) , \end{aligned}$$

where $\varepsilon$ denotes the radiation emissivity coefficient, $\sigma$—the Stefan–Boltzmann constant, $\overrightarrow{n}$ is a vector normal to the surface, and $T_{\infty }$ is the ambient temperature. We solved Eq. (3) using a forward-time central-space (FTCS) scheme^36^ with appropriate space and time resolution to secure stability. Then, we compared the result with the corresponding simulation obtained from the finite element method using Matlab PDE Toolbox^37,38^. Figure 3b presents the temperature distribution on the cylinder radius on the heat source height ($z=h=2.5$ cm, red profile in Fig. 3a). The results are compliant, and the temperature differences decrease when nearing the surface. We also measured the maximum temperature on the cylinder surface after 1 hour of heating, obtaining the 0.07$^{\circ }$C difference between the simulation and theoretical calculations.

### Planar thermal images and initial tomographic reconstruction

The initial reconstruction process employed a series of 2D thermal images composed of synthetically generated data (Fig. 1c). The camera rotating around the object allows for observing temperature distribution on the surface at various angles (360 views with a 1$^{\circ }$ resolution). To obtain individual reconstructions of 2D sections, we employed the backprojection algorithm^39^. In the backprojection reconstruction, each pixel placed at the orientation of a given projection takes a measured projection value. The process repeats for the entire set of angles, and values of corresponding pixels are summed. Finally, the result is divided by the total number of projections. A stack of properly arranged 2D slices finally constructs a 3D volume.

Due to the specifics of infrared radiation, 3D thermal volumes generated using the considered method require further processing to achieve final reconstruction. Areas of increased temperature are located close to the edges of the phantom, so volumetric temperature data is not reliable. Hence, the next stage employs deep learning to improve the accuracy of heat distribution reconstruction within a given medium.

### Convolutional neural network for thermal model reconstruction

**Figure 4:**
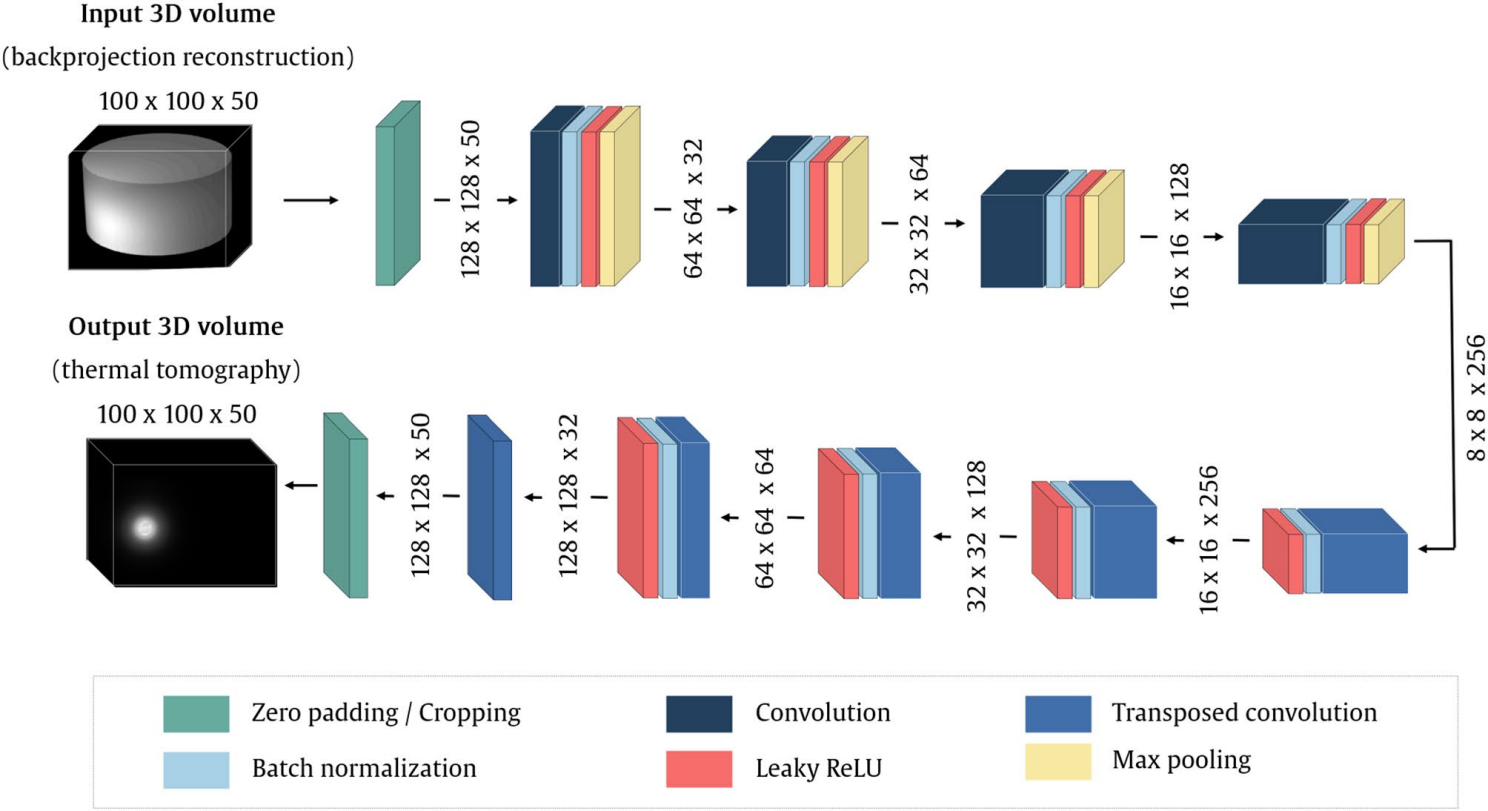
General scheme of the CNN model for thermal model reconstruction.

To reconstruct the temperature distribution, we designed a deep convolutional network of an autoencoder architecture (Fig. 4). The input layer accepts a 100$\times$100$\times$50 volume, padded with zeros to fit the 128$\times$128$\times$50 size. The encoder path consists of four processing blocks. Each involves a 2D convolutional layer (32, 64, 128, 256 filters, 3$\times$3 kernel) followed by batch normalization, leaky rectified linear unit (ReLU), and max pooling (2$\times$2 stride). The decoder path involves four segments, each performing transposed convolutions (256, 128, 64, and 32 filters, respectively, 3$\times$3 kernel, 2$\times$2 stride), batch normalization, and leaky ReLU activation. The reconstruction produces a 100$\times$100$\times$50 volume of thermal tomography data.

The network was trained using the adaptive moment estimation (Adam) optimizer. We randomly partitioned the database of 810 original volumes into the training, validation, and test subsets with an 80$\%$:10$\%$:10$\%$ ratio. To extend the database and increase generalization capabilities, we applied data augmentation in each training epoch by rotating every 3D volume by ten random angles in the axial plane. Hence, a single training sample yielded ten rotated images per epoch. The batch size was set to 32, and the maximum number of epochs to 500. We set both parameters experimentally to gain the highest efficiency and maintain low computational time.

### Evaluation metrics

To assess our framework, we employed several well established metrics. Three were taken from Koutsantonis et al.^16^: normalized mean square error (NMSE), correlation coefficient (CC), being a spatial similarity measure between two images, and peak signal-to-noise ratio (PSNR). From Wang et al.^40^, we adopted the structural similarity index (SSIM).

Finally, we propose a measure for heat source location error (HSLE). It is defined as a distance in millimeters between the reference and the reconstructed heat source midpoint.

## Results

### Tests over a synthetic dataset

**Figure 5:**
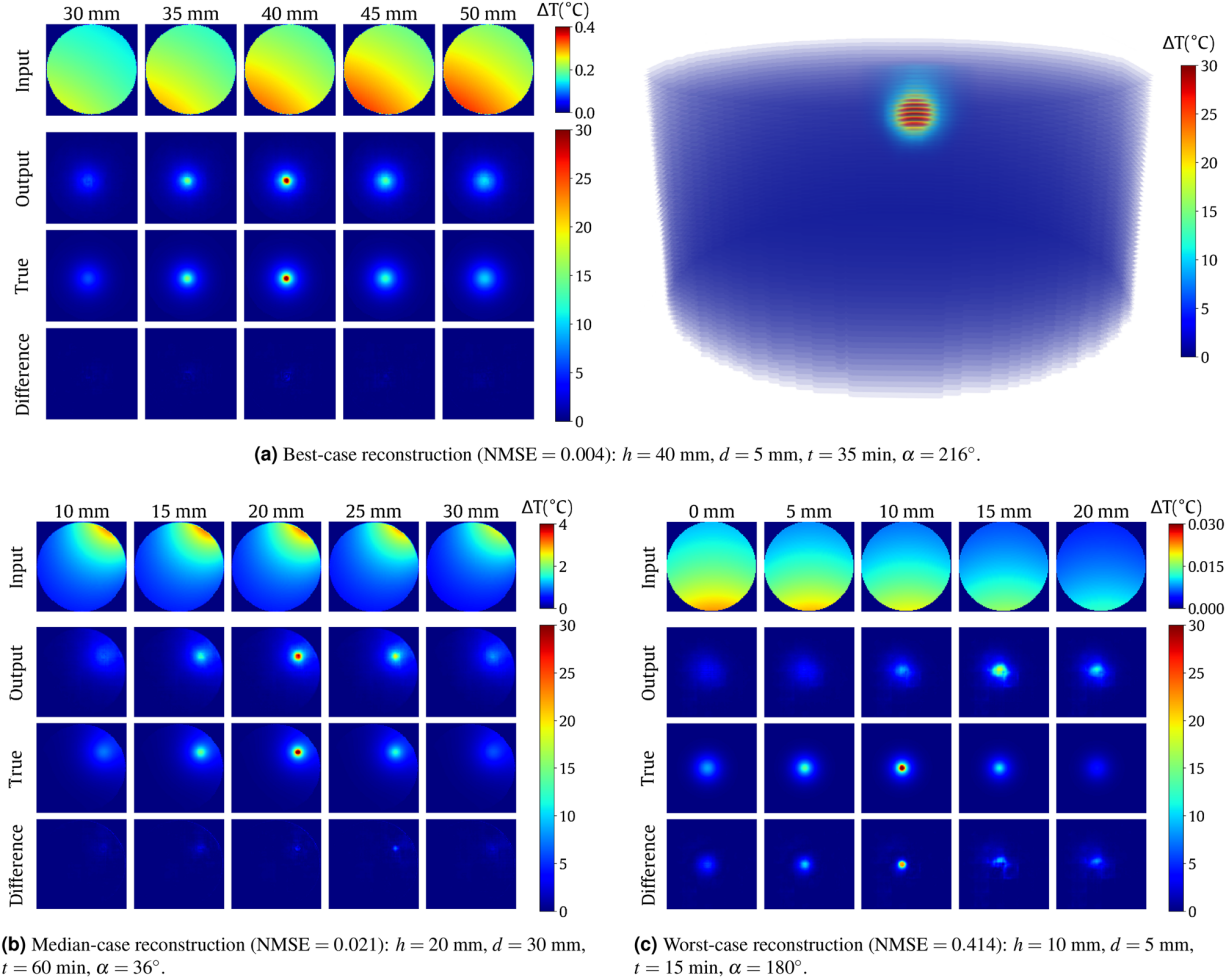
Visualization of the reconstruction results. Top to bottom in each subplot with projections: backprojection slices, final tomographic reconstruction slices, ground-truth slices, the difference between the reconstruction and ground truth (reconstruction error). In each case, the central column contains the heat source center. The location (height) of each slice is given above the column. Volumetric visualization of the reconstruction outcome is given in (**a**) (see Supplementary Video S1).

The original test set coming from a partition described in “Materials and methods” section consisted of 81 unique cases. To extend the testing experiment, we rotated each volume nine times around the main axis with a 36$^{\circ }$ increment (the $\alpha$ angle). This yielded a set of 810 unique test cases. Full testing results are given in Table 2. Figure 5 presents selected visualizations, including the worst-case and best-case reconstructions in terms of NMSE. For best-case recostruction visualization see also Supplementary Video S1.

**Table 2:** Summary of testing the method over a synthetic dataset.

Metrics	Mean ± std. dev.	Median (25–75 percentile)	
NMSE	0.036 ± 0.049	0.021	(0.013–0.035)
CC	0.981 ± 0.026	0.989	(0.981–0.993)
PSNR	43.135 ± 3.386	43.239	(40.856–45.556)
SSIM	0.748 ± 0.114	0.790	(0.697–0.826)
HSLE	1.398 ± 1.146	0.967	(0.689–1.592)

We also estimated the reconstruction time. Most of the time was consumed by the backprojection (113 s per case). The thermal reconstruction using a trained deep model took 0.015 s per case. The tests were performed on a workstation running a 64-bit Windows 10 operating system with an Intel Core i7-8750H CPU, 16 GB DDR4 RAM, and GeForce GTX 1060 GPU with 1280 CUDA cores and 6 GB GDDR5 RAM.

### Experimental validation of the model

We numerically evaluated the assumptions for our model in two experiments involving pork meat of approximately known thermal properties (Table 1). The meat used in the experiments was in no way related to the animal experiments and did not require the approval of the bioethics committee.

**Figure 6:**
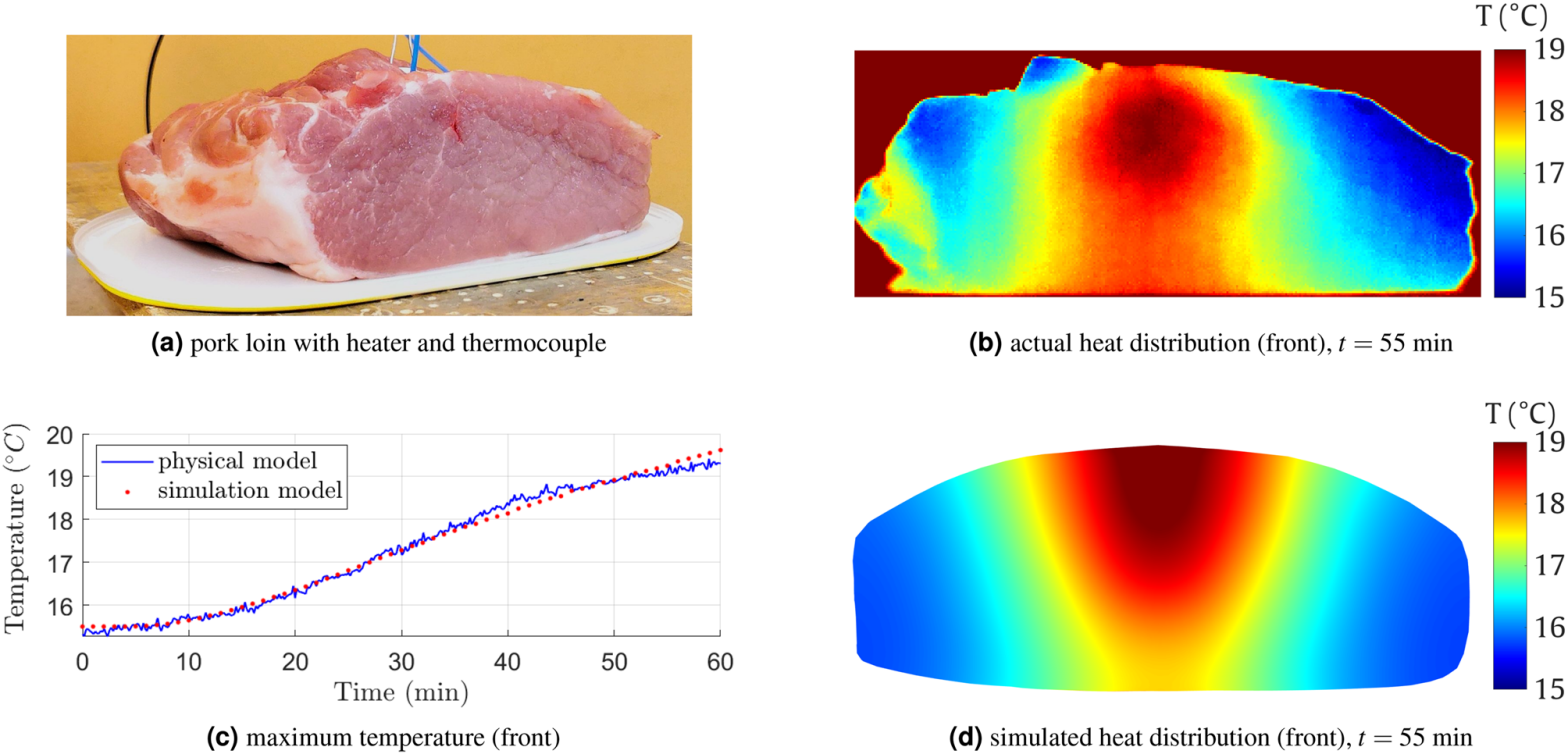
Model evaluation (experiment #1) using a pork loin (**a**), the obtained heat map (**b**), and heat map from the numerical model (**d**) on the front of the loin. The chart (**c**) shows the actual an simulated maximum temperature (front of the loin).

In the first experiment, we used a ca. 0.6 kg pork loin with a resistive heater placed inside (Fig. 6a) at about 3 cm distance from the front (flat side). The measurement stand was surrounded by foam to limit the influence of the surroundings. The heater (a cement 39 $\Omega$, 10 W resistor with external package dimensions of $10\times 12\times 32$ mm) was supplied using a regulated power source. A thermocouple-based digital thermometer controlled its temperature to be 50$^{\circ }$C. During heating, we acquired multiple static thermal images of the front of the loin. Figure 6b presents the thermal map obtained at the front after 55 min of the experiment. The corresponding heat map from our numerical model is shown in Fig. 6d. Figure 6c compares the simulated (red) and actual (blue) maximum temperature on the front of the loin.

**Figure 7:**
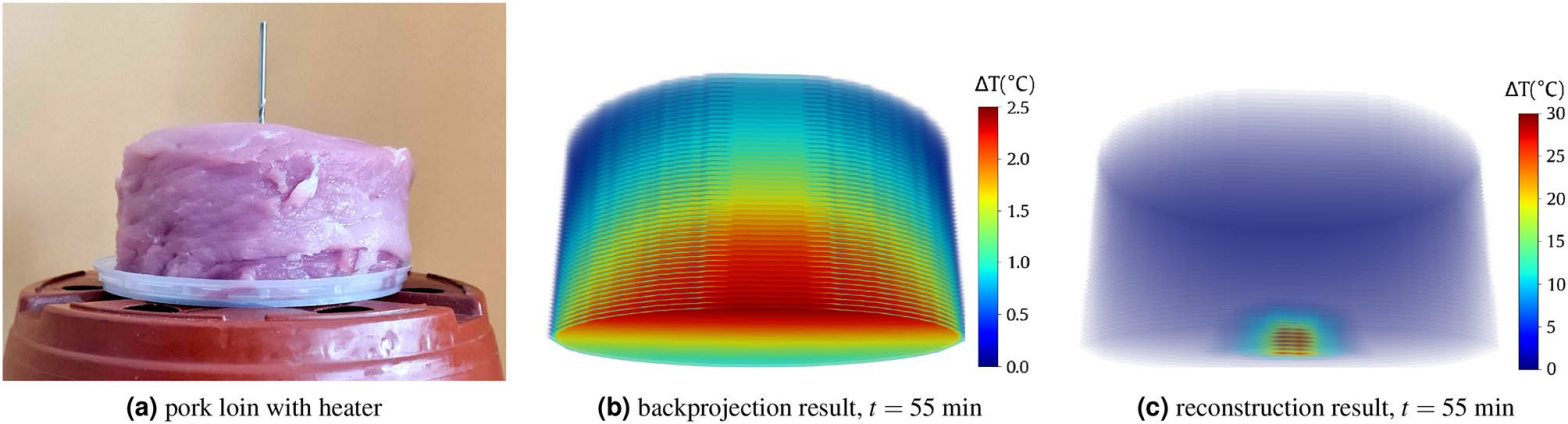
Model evaluation (experiment #2): the cylindrical pork object (**a**), backprojection (**b**) and reconstruction results (**c**).

The second experiment involved pork cut in a cylinder with a ca. 10 cm diameter and 5 cm height (Fig. 7a). The meat was placed on a rotary table with markings every 10$^{\circ }$. The same resistive heater was controlled by an Arduino-based temperature controller, battery-powered, and placed under the model to enable a free rotation of the stand. The heater temperature was first stabilized at 50$^{\circ }$C and then inserted from the bottom into the meat cylinder about $d=15$ mm from the central axis. Then, after 15, 25, 35, 45, and 55 minwe rotated the table and captured a full set of 2D thermal images every 10$^{\circ }$. Figure 7b and c present the example reconstruction in the 55th minute through backprojection and by our trained model, respectively. In the simulation, the maximum temperature $T=48.31^\circ$C ($\Delta T = 30.31^\circ$C) was detected $d=5$ mm from the axis, at $h=5$ mm.

### Experiments with low-temperature heat sources

After verifying our original assumptions over a synthetic dataset and a real object, we prepared additional experiments to check the method’s capability to handle lower temperature differences. The experiments refer to the original setup described in “Materials and methods” section and “Tests over a synthetic dataset” section in terms of both materials and methods. All modifications are depicted in the following sections.

#### Single heat source

First, we took synthetic phantoms with all 81 unique locations of the heat source (“Synthetic dataset” section) and set five different temperatures to produce $\Delta T = 1, 2, 3, 4, 5^{\circ }$C (405 cases). Those relative temperatures can be considered low compared to the original value of $\Delta T = 30^{\circ }$C. During training, we again additionally rotated the phantoms with a $36^{\circ }$ increment to produce 4050 unique cases. This time, we generated a single thermal state per case in terms of heating time. Moreover, since the temperature difference was low, the heat distribution was prepared after 300 min of heating. The validation results are shown in Table 3 using the same metrics as before.

**Table 3:** Summary of testing the method over a synthetic dataset with a single low-temperature heat source.

Metrics	Mean ± std. dev.	Median (25–75 percentile)	
NMSE	0.019 ± 0.010	0.016	(0.012–0.023)
CC	0.978 ± 0.011	0.981	(0.971–0.987)
PSNR	37.354 ± 1.633	37.562	(36.408–38.521)
SSIM	0.952 ± 0.016	0.957	(0.948–0.963)
HSLE	3.597 ± 2.053	3.196	(2.064–4.722)

#### Multiple heat sources

Finally, we extended the low-teperature heat distribution investigation by involving multiple heat sources per phantom. For that purpose, we produced 1000 phantoms with random arrangements of 1–5 heat sources, each of a random 1–5$^{\circ }$C temperature. In each case, the minimum distance between the centers of any two sources was 10 mm (5 mm between their surfaces). Again, we captured a single heat distribution per phantom after 300 min. The validation results of our deep model are presented in the left part of Table 4. We did not determine the HSLE in this case, as it is a metrics designed for single-source setups.

**Table 4:** Summary of testing the method over a synthetic dataset with multiple low-temperature heat sources.

	Original model (four-block CNN)			Extended model (five-block CNN)		
Metrics	Mean ± std. dev.	Median (25–75 percentile)		Mean ± std. dev.	Median (25–75 percentile)	
NMSE	0.022 ± 0.017	0.016	(0.012–0.025)	0.014 ± 0.017	0.008	(0.007–0.012)
CC	0.971 ± 0.020	0.977	(0.968–0.981)	0.980 ± 0.021	0.987	(0.982–0.989)
PSNR	29.579 ± 2.005	29.314	(28.545–30.649)	32.082 ± 2.852	31.834	(30.487–33.661)
SSIM	0.841 ± 0.074	0.849	(0.786–0.900)	0.904 ± 0.040	0.910	(0.879–0.938)

**Figure 8:**
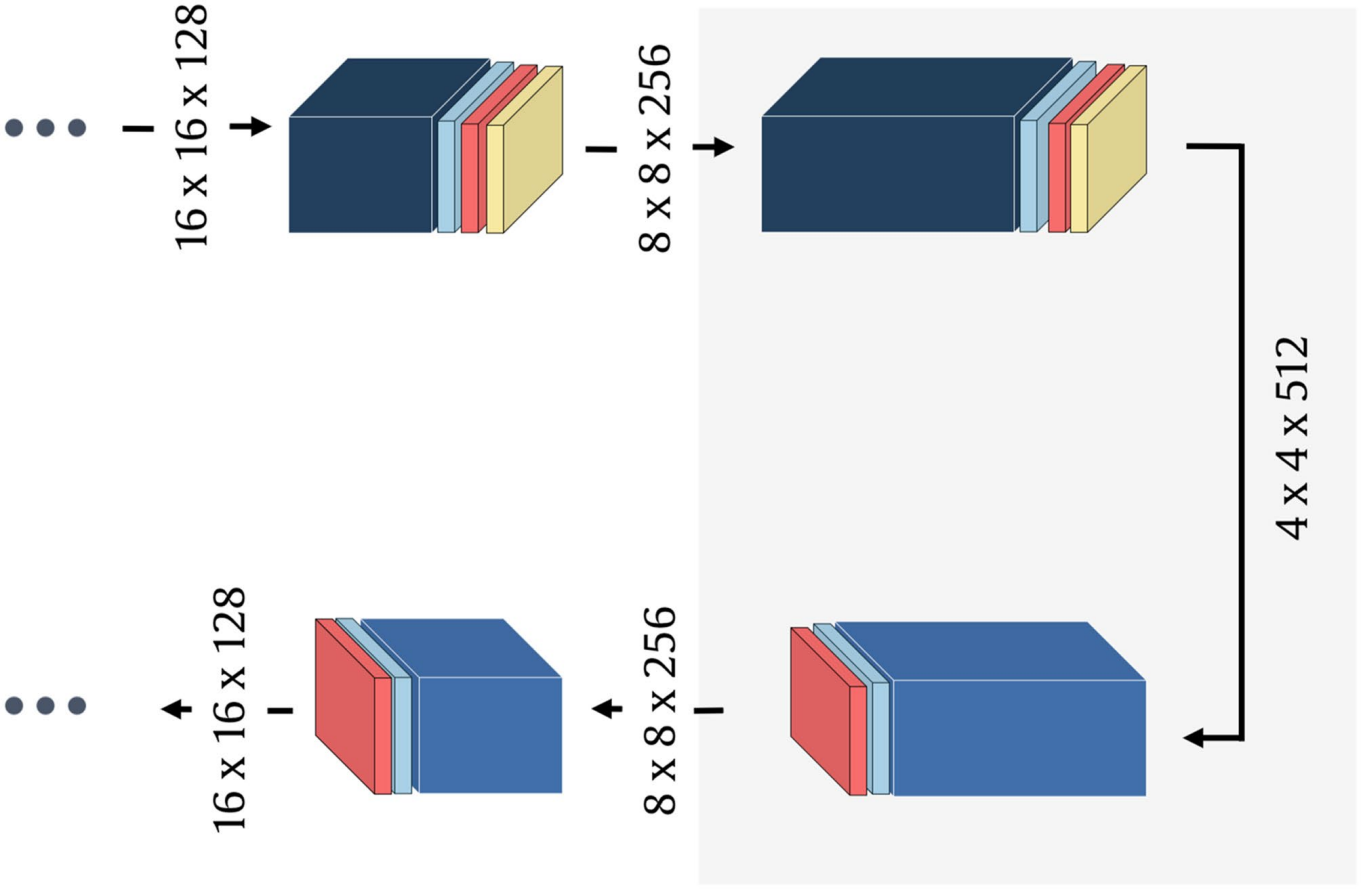
Illustration of the extension of the CNN model from Fig. 4. Added blocks are highlighted with a gray background.

**Figure 9:**
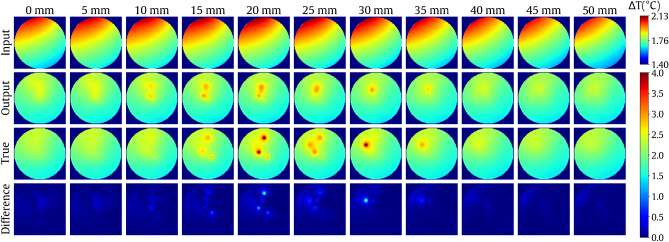
Visualization of the best-case reconstruction ($\text {NMSE}=0.004$) in the multiple-heat-source experiment with a five-block CNN. Top to bottom: backprojection slices, final tomographic reconstruction slices, ground-truth slices, the difference between the reconstruction and ground truth (reconstruction error). The location (height) of each slice is given above the column. For volumetric visualization of the reconstruction outcome, see Supplementary Video S2.

We also verified the multi-source setup using the extended deep model. One processing block was added to both encoder and decoder in the rightmost part form Fig. 4. The five-block encoder produces the 4$\times$4$\times$512 volume (Fig. 8). The results in the right part of Table 4 show the improvement brought by deepening the network. Figure 9 presents the visualization of the best-case reconstructions in terms of NMSE (see also Supplementary Video S2).

## Discussion

The employment of deep learning to 3D tomographic reconstruction from planar thermal images has been the central concept of our study. The results we present support this idea. We started our research in^17^ with the use of backprojection reconstruction over a heated phantom and 2D IR images of a relatively low resolution (36 images with a 10$^{\circ }$ step). Since the IR radiation is not as penetrating as the ionizing one used in, e.g., computed tomography or SPECT, the backprojection has a limited utility. That brought the idea of training a deep model over a series of synthetic data of known properties and heat distributions. Evaluation of the trained model over a synthetic dataset produced at least satisfactory results with low error measures and high spatial similarity between the ground truth and the reconstructed heat distributions.

In all previous studies on thermal tomography, the shape of an object was simplified to a cylindrical form^13,15,16^. We used the same approach here, but the proposed method itself does not have to be limited to one shape. We can prepare synthetic data from any model with specific thermal properties for the training process. Compared to Koutsantonis et al.^16^, our original idea involves a single heat source with a sphere geometry. Contrary to RISE^16^, our results take into account distinct thermal contrast resulting from simulation with varying heating times. Moreover, Koutsantonis’s study addressed semi-transparent medium; we do not set such constraints. Our method allows for a better representation of the temperature distribution in the vicinity of the source compared to the most simplified cases in^16^ (two identical heat sources). We obtained a higher SSIM index, reflecting the visual similarity of the reconstruction and the original image. Our reconstruction is less noisy with higher PSNR values: 43.135 on average than the best-case 33.25 in RISE. Both approaches reliably map the source temperature and location with comparable NMSE and CC levels. We also prove the location mapping correctness using the proposed HSLE metrics with the median below 1 mm.

The most significant errors appeared when the source was $d=0.5$ cm from the central axis of the object, $h=1$ cm from the bottom, after 15 min of heating. Two other cases from the test set with $d=0.5$ cm and after 15 min were reconstructed well (with $h=2.5$ and 3 cm), so the error does not come from the proximity of the source to the central axis. We find the other reasons in a short time to spread the heat towards the external surface and specific temperature distribution at the object edge.

Our thermographic reconstruction simulation was verified in some aspects based on the actual measurements of the physical model. We are fully aware of the limitations of this double experiment (“Experimental validation of the model” section): the location (depth) and dimensions of the object and the heat source (inside the resistor), which are difficult to reproduce in a simplified model accurately, tissue inhomogeneity, or non-insulated base. We are also aware of the limitations of the model: the simplified geometry, homogeneous internal structure, no base. Figures 6b and d show that tissue heterogeneity and shape imperfections contribute to the reconstruction inaccuracy. Moreover, we found some errors in the heat source height estimation. These may result from the influence of the bed on the heat dissipation process, not considered in the simulations. Additional disturbing factors were the ca. 3 min time needed to perform the complete IR acquisition cycle or possible extra heat from the electronic circuitry supplying the heater (under the object). However, taking into account the obtained results and their compliance with the model (Figs. 6c, 7c), despite all the limitations and simplifications, we believe that the method is promising for the appropriate reconstruction of real-object volumetric temperature distribution.

In this pilot study on CNN-based reconstruction, we mainly focused on simulating a single heat source of a basic shape (a spherical heat source). Except for the physical model experiment, we did not investigate models different from cylindrical. The natural further development direction is to investigate more complex forms and shapes through model training and validation. We believe that the deep model can follow such structures, perhaps through some superposition of learned patterns. Diversification of the training and test sets by introducing rotations of the original data supported this assumption: the network performed equally well regardless of the rotation angle. In “Experiments with low-temperature heat sources” section, we aimed at verifying more complex tasks in terms of heater temperature and the number of heat sources. Lowering the relative source temperature $\Delta T$ from 30 to 5$^{\circ }$C and less did not result in a decreased performance. Hence, the model can produce robust reconstruction regardless of $\Delta T$ with the applied assumptions. We also made another step forward by randomly placing up to five low-temperature heat sources in a single phantom. The obtained results are promising (Table 4, Fig. 8). The network trained with multi-source phantoms reconstructs the temperature distribution affected by the mutual influence of several sources. The exact temperature of particular sources themselves remains an issue. However, in single-source testing cases, both the heater temperature and location are estimated correctly, so the properties of the two previous models are retained. Extending the model with additional convolution blocks increased its capacity, thanks to which it was possible to obtain better results while increasing the complexity of the data set. The reconstructions are visually smoother, which is also reflected in improved PSNR values.

So far, our framework involves the backprojection algorithm as an intermediate step between IR image acquisition and thermal reconstruction. However, we consider avoiding it in our future research, also due to significant time consumption. That would enable reconstructing the heat distribution based directly on a series of planar IR images of the object. However, such an approach will require studies on the optimal arrangement of IR images in the deep model input in terms of spatial relationships.

## Conclusion

Our study introduces the artificial intelligence concept into a relatively poorly explored field of thermal tomography. Our results show the capability of a deep model to reconstruct the volumetric temperature distribution from a sequence of planar thermal images of an object containing variable number of heat sources. We find the idea promising and share it with the scientific community with some plans for developing the method and models.

## Supplementary Information

Supplementary Video 1.

Supplementary Video 2.
