# Supplementary figures and images for: Tomographic reconstruction from planar thermal imaging using convolutional neural network
Source: Sci Rep. 2022 Feb 11;12:2347. doi: 10.1038/s41598-022-06076-z (PMC8837619; doi:10.1038/s41598-022-06076-z)

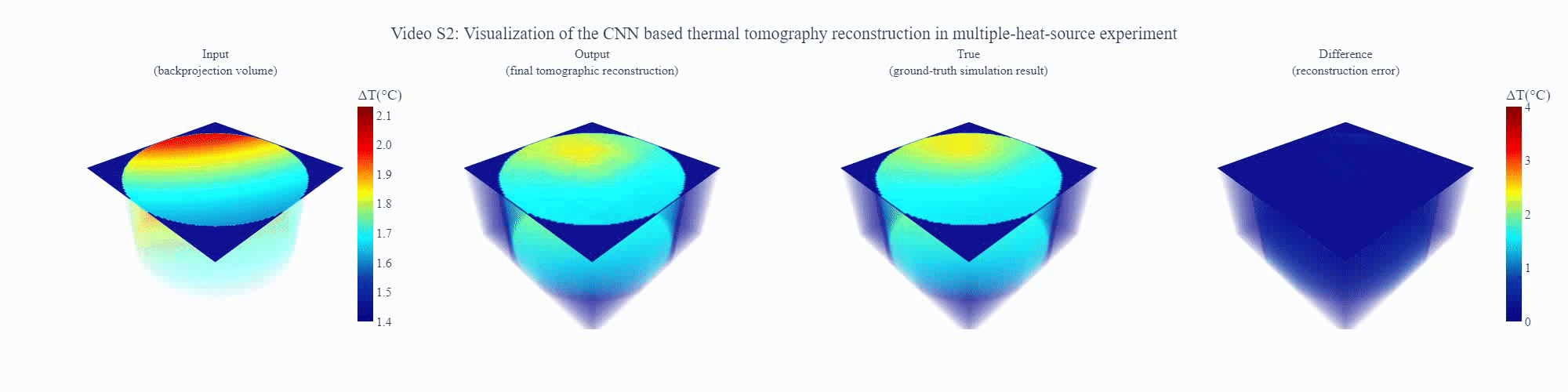

Supplement: Supplementary file 1 — Supplementary Video 1. [file 41598_2022_6076_MOESM1_ESM.gif]

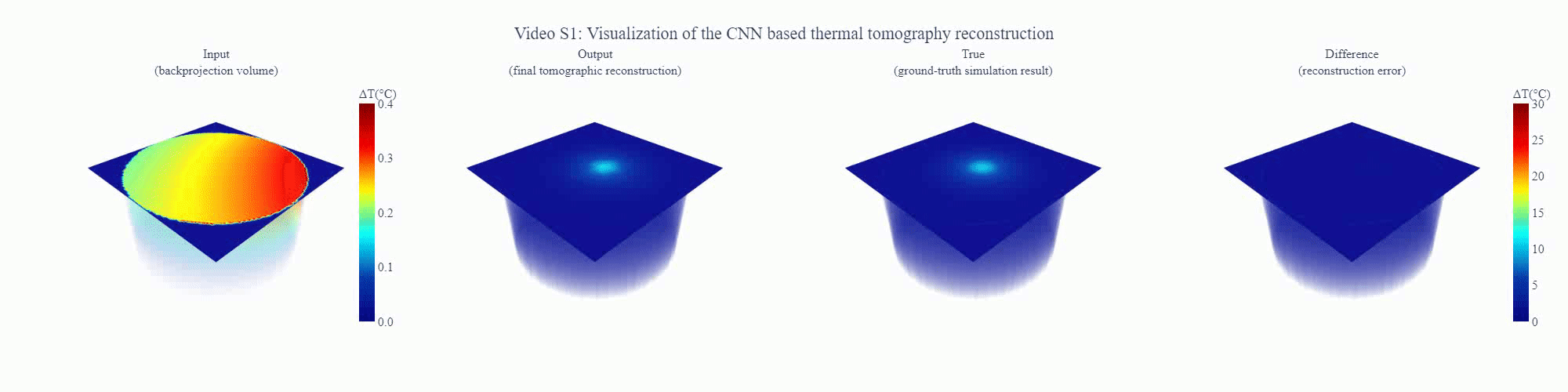

Supplement: Supplementary file 2 — Supplementary Video 2. [file 41598_2022_6076_MOESM2_ESM.gif]
